# Role of lipoxygenase products in the effects of angiotensin II in the isolated aorta and perfused heart of the rat

**DOI:** 10.1155/S0962935195000676

**Published:** 1995-11

**Authors:** J. P. M. Dam, E. van den Worm, W. Vleeming, M. J. Post, A. J. Porsius, J. Wemer

**Affiliations:** 1 Utrecht University Faculty of Pharmacy Department of Pharmacoepidemiology & Pharmacotherapy P.O. Box 80 082 Utrecht 3508 TB The Netherlands; 2 National Institute of Public Health and Environmental Protection P.O. Box 1 Bilthoven 3720 BA The Netherlands; 3 University Hospital Utrecht Department of Cardiology Heart Lung Institute P.O. Box 85 500 Utrecht 3508 GA The Netherlands; 4 pharma Bio-Research Science Park P.O. Box 200 Zuidlaren 9470 AE The Netherlands; 5 University Hospital Utrecht Department of Cardiology Heart Lung Institute G02.523, P.O. Box 85 500 Utrecht 3508 GA The Netherlands

## Abstract

The objective of this study was to determine whether arachidonate metabolites are involved in the vasoconstrictive effects of angiotensin II in rats. In the isolated perfused heart, dexamethasone (4 mg/kg) significantly suppressed the maximal decreases in coronary flow induced by angiotensin II and vasopressin (reference drug). In the heart, the nonselective lipoxygenase inhibitor nordihydroguaiaretic acid (NDGA, 1 μM) markedly suppressed the angiotensin II-induced decreases in coronary flow. NDGA (10 μM) inhibited both angiotensin II- and methoxamine- (reference drug) induced contractions in aortic rings with (in the presence of L-NAME) and without endothelium. In the heart, the leukotriene synthesis inhibitor MK-886 (0.3 μM) significantly reduced the maximal effects to angiotensin II, but the leukotriene antagonist FPL 55712 (0.1 and 0.3 μM) had no effect. We conclude that in the isolated perfused rat heart angiotensin II-induced decreases in coronary flow are in part mediated by Hpoxygenase products, which might be derived from the 5-Hpoxygenase pathway, but are probably not leukotrienes. Furthermore, endothelium independent Hpoxygenase products mediate part of the contractile responses to angiotensin II in the isolated rat aorta.


Mediators of Inflammation 4, 417–425 (1995)

### Role of lipoxygenase products in the effects of angiotensin II in the isolated aorta and perfused heart of the rat

J. P. M. Dam,*[1,CA] E. van den Worm,[1]

W. Vleeming,[2] M. J. Post,[3] A. J. Porsius[1] and

J. Wemer[4]

[1]Utrecht University, Faculty of Pharmacy, Department of Pharmacoepidemiology & Pharmacotherapy, P.O. Box 80 082, 3508 TB Utrecht; [2]National Institute of Public Health and Environmental Protection, P.O. Box 1, 3720 BA Bilthoven; [3]University Hospital Utrecht, Department of Cardiology, Heart Lung Institute, P.O. Box 85 500, 3508 GA Utrecht and [4]Pharma Bio-Research, Science Park, P.O. Box 200, 9470 AE Zuidlaren, the Netherlands

*Present address: University Hospital Utrecht, Department of Cardiology, Heart Lung Institute, G02.523, P.O. Box 85 500, 3508 GA Utrecht, the Netherlands.

[CA]Corresponding Author

**THE objective of this study was to determine whether arachidonate metabolites are involved in the vasoconstrictive effects of angiotensin II in rats. In the isolated perfused heart, dexamethasone (4 mg/kg) significantly suppressed the maximal decreases in coronary flow induced by angiotensin II and vasopressin (reference drug). In the heart, the nonselective lipoxygenase inhibitor nordihydroguaiaretic acid (NDGA, 1 μM) markedly suppressed the angiotensin II-induced decreases in coronary flow. NDGA (10 μM) inhibited both angiotensin II- and methoxamine-(reference drug) induced contractions in aortic rings with (in the presence of L-NAME) and without endothelium. In the heart, the leukotriene synthesis inhibitor MK-886 (0.3 μM) significantly reduced the maximal effects to angiotensin II, but the leukotriene antagonist FPL 55712 (0.1 and 0.3 μM) had no effect. We conclude that in the isolated perfused rat heart angiotensin II-induced decreases in coronary flow are in part mediated by lipoxygenase products, which might be derived from the 5-lipoxygenase pathway, but are probably not leukotrienes. Furthermore, endothelium independent lipoxygenase products mediate part of the contractile responses to angiotensin II in the isolated rat aorta.**

**Key words:** Angiotensin II, Aorta, Endothelium, Heart, Lipoxygenase, Rat

### Introduction

Vascular angiotensin II is involved in the long-term regulation of blood vessel function and structure and is a major pathophysiological factor in hypertension, atherosclerosis and restenosis.[1] There is accumulating evidence for the presence of a complete renin–angiotensin system in the heart, and a functional role has been suggested.[2–5] Locally generated angiotensin II could be of importance in the regulation of coronary flow, as has for instance been demonstrated in patients with microvascular angina (Syndrome X).[6] Furthermore, angiotensin II has a direct effect on protein synthesis, which contributes to cardiac hypertrophy.[7]

It is well known that angiotensin II exerts its effects through stimulation of inositol phospholipid metabolism. Angiotensin II activates phospholipase C, which hydrolyses phosphatidylinositol biphosphate into inositol triphosphate and 1,2-diacylglycerol.[8] In addition, it has been demonstrated recently that 12-lipoxygenase activation plays a key role in, e.g. angiotensin II-induced vascular smooth muscle cell hypertrophy.[9] Other studies have shown that arachidonate metabolites contribute to various responses to angiotensin II, including inflammatory, contractile and secretory actions.[10–16] This paper deals with the contribution of arachidonate metabolites to the vasoconstrictive effect of angiotensin II in the coronary circulation and aorta of the rat. If arachidonate metabolites play a role in coronary constrictions evoked by angiotensin II, it could be hypothesized that drugs inhibiting arachidonate metabolism have a beneficial effect in the prevention of angiotensin II-induced vasospasms, that can lead to angina pectoris or hypertension. To assess the contribution of arachidonate metabolites to angiotensin II-induced responses, we tested several drugs that interfere with arachidonate metabolites in isolated Langendorff perfused rat hearts and isolated aortas. To study whether arachidonate dependent effects were selective for angiotensin II we also tested methoxamine and vasopressin as agonists, which like angiotensin II exert their effects through stimulation of inositol phospholipid metabolism. We examined the cyclo-oxygenase pathway in the effects of angiotensin II by using the cyclo-oxygenase inhibitor indomethacin, the lipoxygenase pathway was studied by using the non-selective lipoxygenase (and cyclo-oxygenase) inhibitor nordihydroguaiaretic acid (NDGA), the

© 1995 Rapid Science Publishers

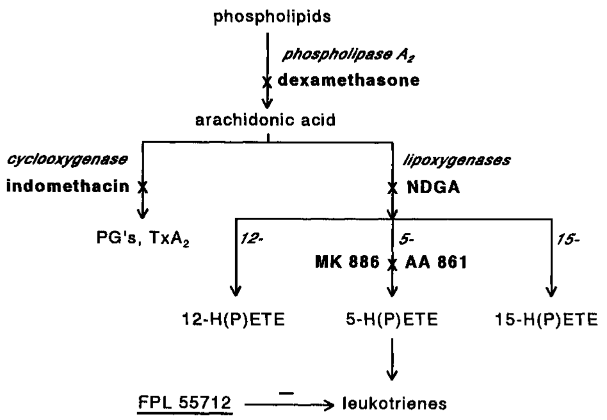

FIG. 1. Scheme of metabolism of arachidonic acid via the cyclooxygenase and lipoxygenase pathway. Enzyme inhibitors are given in bold type. **NDGA**, nordihydroguaiaretic acid; PGs, prostaglandins; $TxA_2$, thromboxane $A_2$; H(P)ETE, hydro(pero)xyeicosatetraenoic acid. The leukotriene antagonist FPL 55712 is underlined.

5-lipoxygenase inhibitor 2,3,4,5-tri-methyl-6-(12-hydroxy-5,10-dodecadiynyl)-1,4-ben-zoquinone (AA 861), the leukotriene synthesis inhibitor 3-[1-(*p*-chlorobenzyl)-5-(isopropyl)-3-*tert*-butylthioindol-2-yl]-2,2-dimethylpropanoic acid (MK-886) and the leukotriene antagonist sodium 7-[3-(4-acetyl-3-hydroxy-2-pro-pylphenoxy)-2-hydroxy-propoxy]-4-oxo-8-propyl-4*H*-1-benzopyran-2-carboxylate (FPL 55712). Further-more, dexamethasone was used to inhibit the production of arachidonic acid via the phospholipase $A_2$ pathway (Fig. 1). Losartan (DuP 753) was used to determine whether the angiotensin II-induced effects were mediated by the angiotensin II receptor subtype-1. In addition, to study the origin of arachidonate metabolites, we performed experiments in the aorta in the presence (with the nitric oxide synthase inhibitor $N^G$-nitro-L-arginine methyl ester (L-NAME)) and absence of endothelium.

### Materials and Methods

*Animals:* In this study we used 196 male Wistar rats (180–260 g). Food and water were allowed *ad libitum*. After heparinization (1000 IU/kg i.p.) rats were killed by decapitation.

*Perfusion of the isolated heart:* The heart of each rat was rapidly excised and immediately mounted for perfusion by the Langendorff technique, using constant pressure of 80 cm water.[17] The perfusion fluid was Krebs–Ringer (pH = 7.4) containing (expressed as mM) NaCl, 128; KCl, 4.7; $MgCl_2$, 0.6; $NaH_2PO_4$, 0.4; $NaHCO_3$, 27; $CaCl_2$, 1.3; and glucose 11. The Krebs–Ringer solution was kept at 32°C and equilibrated with 95% $O_2$ and 5% $CO_2$ throughout the experiment. Coronary flow was measured with an extracorporeal flowsensor (Skalar Medical, Delft, the Netherlands) and recorded on a Gould recorder. In the heart, left ventricular pressure was measured by a water filled balloon (HSE, Freiburg, Germany) and recorded on a Gould recorder. The diastolic left ventricular pressure was adjusted between 5 and 10 mm Hg. The heart was electrically driven at a frequency of 5 Hz (32°C) and equilibrated for 30 min. The experiments were performed at 32°C, since at 37°C some hearts escape pacing. After equilibration, dose–response curves were obtained with vasopressin ($10^{-15}$ – $10^{-8}$ moles) and angiotensin II ($10^{-16}$ – $10^{-8}$ moles). The agonists (0.1 ml bolus injections) were administered directly into the perfusion stream just proximal to the heart. After each dose a stable new baseline was reached before the next dose was given.

*Preparation of aortic rings:* The thoracic aorta was rapidly excised and immersed in Krebs–Ringer solution of the following composition (mM): NaCl, 118; KCl, 5.9; $MgSO_4.7H_2O$, 1.2; $CaCl_2.2H_2O$, 2.5; $NaH_2PO_4.H_2O$, 1.2; $NaHCO_3$, 24.9; $Ca_2EDTA$, 0.026; and glucose 11.1 (pH = 7.4). After cleaning the aorta from fat and connective tissue, it was cut into rings of 2–3 mm length. In some rings, the endothelial layer was removed by inserting the tip of a forceps in the lumen of the rings and gently rolling the rings on moistened filter paper. The rings were suspended between two stirrups in 10 ml organ chambers filled with oxygenated (95% $O_2$/5% $CO_2$) Krebs–Ringer solution. The temperature was maintained at 37°C. Changes in force were measured with an isometric force transducer (Harvard Bioscience) and recorded by computer. The aortic rings were set at an initial resting tension of 2 g. After 60 min of equilibration, during which the Krebs–Ringer solution was replaced every 15 min, the rings were challenged twice with 100 mM KCl, with an interval of 35 min, wherein the resting tensions were readjusted to 2 g. The absence of endothelium was confirmed by the lack of acetylcholine (1 μM) induced relaxations in rings precontracted with 0.1 μM phenylephrine.

*Aorta experiments:* Cumulative concentration–response curves for angiotensin II (0.1–100 nM) and methoxamine (0.01–100 μM) were constructed in aortic rings with and without endothelium after 30 min of incubation with 0.1% ethanol, NDGA (1, 3 or 10 μM), AA 861 (10 μM) or after incubation with 10 μM indomethacin (part of the Krebs–Ringer solution). The experiments with 0.1% ethanol and NDGA (3 and 10 μM) were also performed with vasopressin (1–1000 nM).

Experiments with endothelium were conducted with and without 100 μM L-NAME added to the incubation medium.

Finally, concentration–response curves for angiotensin II (0.1–1000 nM) were constructed after 30 min of incubation with losartan (0.01, 0.1 and 1 μM) in aortic rings with endothelium in the presence of 100 μM L-NAME or in rings without endothelium.

*Drugs and drug solutions:* Angiotensin II (acetate salt), indomethacin, NDGA, methoxamine HCl, phenylephrine HCl, acetylcholine HCl, L-NAME and sodium nitroprusside (SNP) were obtained from Sigma Chemical (St. Louis, MO, USA). AA 861 (2,3,4,5-trimethyl-6-(12-hydroxy-5,10-dodecadiynyl)-1,4-benzoquinone) was obtained from Takeda Chem. Ind. Ltd. (Osaka, Japan). Losartan (DuP 753) was a gift from du Pont de Nemours & Company (Wilmington, Del., USA). Heparin was from Leo (Weesp, the Netherlands) and sodium pentobarbitone was from Ceva (Paris, France). Vasopressin (Vasopressin-Sandoz: lypressin) was a gift from Sandoz, Wander Pharma (Uden, the Netherlands). FPL 55712 (sodium 7-[3-(4-acetyl-3-hydroxy-2-propylphenoxy)-2-hydroxypropoxy]-4-oxo-8-propyl-4*H*-1-benzopyran-2-carboxylate) was a gift from Fisons Pharmaceuticals (Loughborough, England). MK-886 was a gift from Merck Frosst (Quebec, Canada) and dexamethasone phosphate (decadron) was obtained from O.P.G. (Utrecht, the Netherlands). All other chemicals were of analytical grade (Merck, Darmstadt, Germany).

Unless otherwise specified, drugs were dissolved in saline. Dexamethasone phosphate (4 mg/kg) and saline (2 ml/kg) were administered into the tail vein 5.5 h prior to testing. NDGA, AA 861, and MK-886 were dissolved in ethanol. For the aorta experiments NDGA and AA 861 were diluted with saline (final concentration ethanol, $\leqslant$ 0.1%). For the heart experiments NDGA, AA 861 and MK-886 were diluted to the appropriate concentration in the perfusion fluid (final concentration ethanol, $\leqslant$ 0.005%). FPL 55712, SNP and losartan were dissolved in bidistilled water and diluted to the appropriate concentration in the perfusion fluid. Indomethacin was dissolved in 4.54% $NaHCO_3$ and then added to the Krebs–Ringer solution (final concentration $NaHCO_3$: heart, 27 mM; aorta, 24.9 mM). Vasopressin was diluted with saline. Solutions were freshly prepared every day and kept on ice and protected from light.

In experiments using isolated hearts, NDGA, AA 861, MK-886, FPL 55712, SNP, losartan (and indomethacin) were already present in the perfusion fluid before mounting the hearts, during the equilibration time of 30 min and during the construction of the dose–response curves. One dose–response curve was constructed per heart in all treatment and control groups.

*Data analysis:*

*Heart.* When baseline coronary flow was significantly enhanced by drugs (NDGA, AA 861, MK-886 or FPL 55712), we used control groups with SNP in sufficient concentration to reach a baseline coronary flow that was equal to the baseline coronary flow in the drug groups. These control groups with SNP were added to exclude the possibility that the enhancing effects of the drugs on baseline coronary flow might obscure the possible effects of these drugs on angiotensin II-induced constrictions. Baseline coronary flow values were analysed by Student's *t*-test or ANOVA followed by Duncan's Range Test for multiple comparison.

Coronary vasoconstriction induced by angiotensin II and vasopressin is expressed as the percentage reduction of baseline coronary flow. Dose–response curves of angiotensin II and vasopressin were analysed by MANOVA with a repeated measures design.

*Aorta.* Contractions are expressed as percentage of the maximal contraction of 100 mM KCl. Concentration–response curves for angiotensin II, methoxamine and vasopressin were analysed by MANOVA using a repeated measures design.

*Heart and aorta.* A $p$-value $< 0.05$ was considered statistically significant. Data are expressed as means $\pm$ S.E.M. In the figures, a significant difference between the entire dose/concentration–response curves is indicated with an asterisk.

### Results

*Isolated Langendorff perfused heart:*

*Baseline values.* Baseline coronary flow values for all groups are shown in Table 1. Since NDGA, AA 861, MK-886 and FPL 55712 enhanced baseline coronary flow, control groups were used with SNP 0.01–0.03 μM to reach a similar baseline coronary flow.

*Angiotensin II and vasopressin effects.* Angiotensin II induced a dose-dependent decrease in coronary flow with a $pD_2$ of 11.5 $\pm$ 0.1 and an $E_{max}$ of 55.9 $\pm$ 2.4% ($n = 8$) (Fig. 2). Losartan (0.01–10 μM) reduced the angiotensin II-induced decreases in coronary flow in a concentration dependent manner. The effect of losartan $10^{-5}$ M on the response to angiotensin II is shown in

Table 1. Baseline coronary flow (CF) and left ventricular pressure (LVP) values

| | $n$ | CF (ml/min) | LVP (mmHg) |
|---|---|---|---|
| **Angiotensin II** | | | |
| Control: Krebs-Ringer | 8 | $5.7 \pm 0.3$ | $77.1 \pm 7.1$ |
| Losartan 10 μM | 8 | $5.8 \pm 0.6$ | $74.5 \pm 4.6$ |
| Indomethacin 10 μM | 7 | $6.4 \pm 0.5$ | $73.1 \pm 4.9$ |
| Saline 2 ml/kg −5.5 h | 5 | $6.9 \pm 0.2$ | $66.6 \pm 5.6$ |
| Dexamethasone 4 mg/kg −5.5 h | 5 | $6.8 \pm 0.3$ | $81.0 \pm 6.4$ |
| Control: SNP 0.01 μM | 7 | $7.7 \pm 0.5$ | $82.1 \pm 3.1$ |
| NDGA 1 μM | 8 | $7.2 \pm 0.2$ | $74.1 \pm 4.8$ |
| MK-886 0.3 μM | 5 | $7.7 \pm 0.1$ | $69.2 \pm 8.7$ |
| FPL 55712 0.1 μM | 8 | $7.0 \pm 0.3$ | $71.9 \pm 4.0$ |
| FPL 55712 0.3 μM | 8 | $6.9 \pm 0.2$ | $67.5 \pm 7.0$ |
| Control: SNP 0.03 μM | 6 | $8.5 \pm 0.3$ | $65.3 \pm 5.6$ |
| AA 861 5 μM | 8 | $8.6 \pm 0.6$ | $55.9 \pm 5.3$ |
| AA 861 5 μM and indomethacin 10 μM | 6 | $8.3 \pm 0.3$ | $45.8 \pm 3.9$* |
| **Vasopressin** | | | |
| Control: Krebs-Ringer | 6 | $6.0 \pm 0.2$ | $88.5 \pm 2.3$ |
| Saline 2 ml/kg −5.5 h | 6 | $5.8 \pm 0.3$ | $65.5 \pm 4.8$ |
| Dexamethasone 4 mg/kg −5.5 h | 6 | $6.3 \pm 0.5$ | $86.5 \pm 2.0$* |
| Control: SNP 0.01 μM | 8 | $8.0 \pm 0.4$ | $87.4 \pm 3.2$ |
| NDGA 1 μM | 8 | $7.2 \pm 0.1$ | $77.9 \pm 2.7$* |
| AA 861 5 μM | 8 | $7.9 \pm 0.2$ | $63.6 \pm 5.2$* |
| MK-886 0.3 μM | 5 | $8.3 \pm 0.1$ | $83.8 \pm 3.8$ |

Drug groups were compared with their respective control (e.g. indomethacin *vs.* Krebs-Ringer, NDGA 1 μM *vs.* SNP 0.01 μM and dexamethasone *vs.* saline pretreatment). *significant ($p < 0.05$) difference between drug(s) and respective control (Krebs-Ringer, SNP 0.01 μM, SNP 0.03 μM or saline 2 ml/kg, −5.5 h).

Fig. 2. The $pA_2$ value calculated for losartan in the isolated perfused heart was 8.5. Also with vasopressin a dose-dependent decrease in coronary flow was observed that was larger than for angiotensin II ($pD_2$, $10.0 \pm 0.1$; $E_{max}$, $74.2 \pm 5.1\%$) (data not shown). The effect of angiotensin II on left ventricular pressure was marginal, whereas the left ventricular pressure was decreased at high vasopressin concentrations (1 and 10 nM) (data not shown).

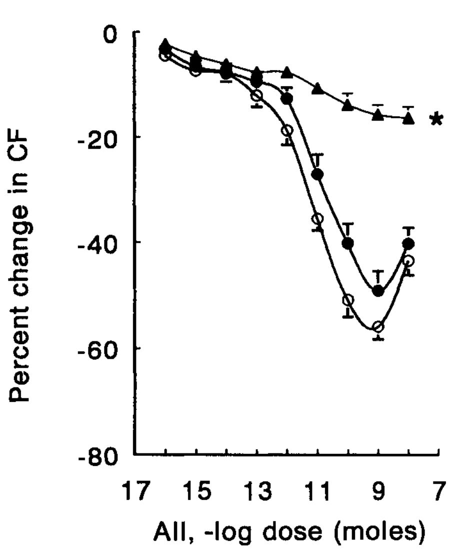

FIG. 2. Effects of bolus injections of angiotensin II (AII) on coronary flow (CF) in isolated perfused rat hearts in the absence (○) and presence of 10 μM losartan (▲) or 10 μM indomethacin (●). Results are means ± S.E.M. of six to eight experiments.

*Modulation by dexamethasone.* After pretreatment with 4 mg/kg dexamethasone for 5.5 h, the maximal angiotensin II- and vasopressin-induced reductions in coronary flow were significantly reduced from $53.7 \pm 3.8\%$ ($n = 5$, saline pretreated rats) to $40.5 \pm 3.6\%$ ($n = 5$; Fig. 3, left panel) and from $74.9 \pm 7.2\%$ ($n = 6$, saline pretreated rats) to $57.5 \pm 3.6\%$ ($n = 6$; Fig. 3, right panel), respectively.

*Modulation by indomethacin, NDGA and AA 861.* Indomethacin (10 μM) did not affect the flow reduction by angiotensin II ($pD_2$, $11.1 \pm 0.1$; $E_{max}$, $49.1 \pm 3.7\%$, $n = 7$, N.S.) (Fig. 2). NDGA (1 μM) significantly reduced the maximal constriction to angiotensin II from $48.4 \pm 3.1\%$ ($n = 7$) to $23.9 \pm 2.6\%$ ($n = 8$; Fig. 4, left panel). NDGA (1 μM) also had a significant inhibitory effect on the responses to vasopressin (Fig. 4, right panel).

The 5-lipoxygenase inhibitor AA 861 (5 μM) did not significantly reduce the responses to angiotensin II (Fig. 5, left panel) and vasopressin (Fig. 5, right panel) (angiotensin II, 30 nM SNP; vasopressin, 10 nM SNP). To test whether indomethacin induced shunting of arachidonate metabolism through the lipoxygenase pathway, we combined indomethacin with AA 861. The combination of AA 861 (5 μM) and indomethacin (10 μM) significantly reduced maximal angiotensin II-induced constrictions from $42.9 \pm 3.8\%$ ($n = 6$; control, 30 nM SNP) to $22.3 \pm 3.4\%$

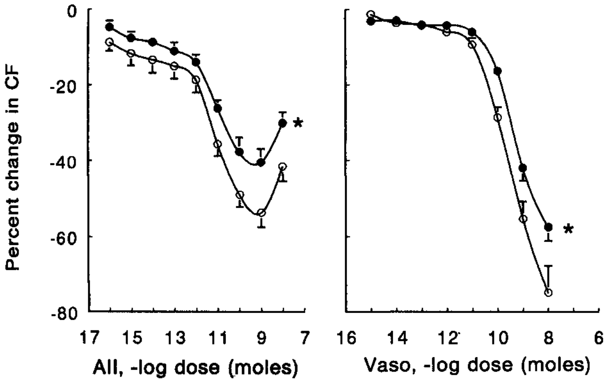

FIG. 3. Effects of bolus injections of angiotensin II (AII, left panel) and vasopressin (VASO, right panel) on coronary flow (CF) in isolated perfused rat hearts after pretreatment with saline (2 ml/kg) (○) or dexamethasone 4 mg/kg (●) 5.5 h prior to testing. Results are means ± S.E.M. of five to six experiments. Asterisks indicate significant ($p < 0.05$) difference between curves.

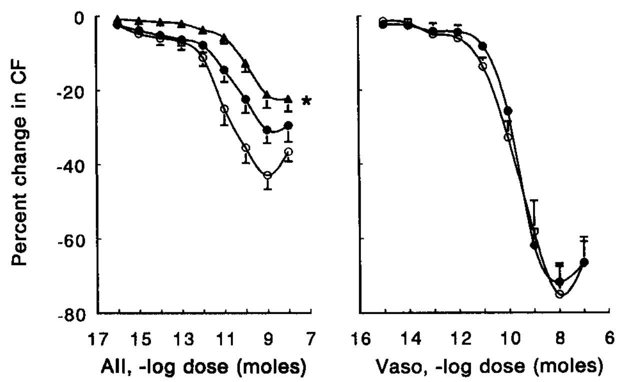

FIG. 5. Effects of bolus injections of angiotensin II (AII, left panel) and vasopressin (VASO, right panel) on coronary flow (CF) in isolated perfused rat hearts in the presence of 30 nM (left panel), 100 nM (right panel) sodium nitroprusside (○), 5 µM AA 861 (●) or the combination of 5 µM AA 861 and 10 µM indomethacin (▲). Results are means ± S.E.M. of six to eight experiments. Asterisk indicates significant ($p < 0.05$) difference between drugs and SNP.

($n = 6$; Fig. 5, left panel). No effect of ethanol 0.005% was observed on angiotensin II-induced reductions in coronary flow (data not shown).

*Modulation by MK-886 and FPL 55712.* Pretreatment with 0.3 µM MK-886 had a significant inhibitory effect on the maximal angiotensin II-induced decreases in coronary flow (Fig. 6, left panel), but not on the vasopressin-induced decreases in coronary flow compared to the 0.01 µM SNP control group (Fig. 6, right panel). FPL 55712 (0.1 and 0.3 µM) had no effect on the angiotensin II-induced decreases in coronary flow (Fig. 6, left panel).

### *Isolated aorta:*

*Effects of NDGA on angiotensin II-, methoxamine-, and vasopressin-induced contractions in aortic rings without endothelium.* In the presence of 0.1% ethanol, angiotensin II induced concentration dependent contractions in aortic rings without endothelium (Fig. 7a). The maximal contractions induced by angiotensin II were inhibited by 3 and 10 µM NDGA in a concentration dependent manner. Pretreatment with 10 µM NDGA caused a significant depression of the contractions to angiotensin II compared to control: $E_{max}$, 61.3 ± 12.4% ($n = 6$) and 157.5 ± 6.8% ($n = 6$), respectively (Fig. 7a). For methoxamine-induced contractions the maximal effect was only slightly reduced by NDGA (from 175.3 ± 5.3% ($n = 6$) to 145.9 ± 9.3% ($n = 7$)), while a pronounced rightward shift of the concentration–response curve was observed (Fig. 7b). The vasopressin response curve was less pronounced shifted to the right by NDGA than that of methoxamine, and like methoxamine

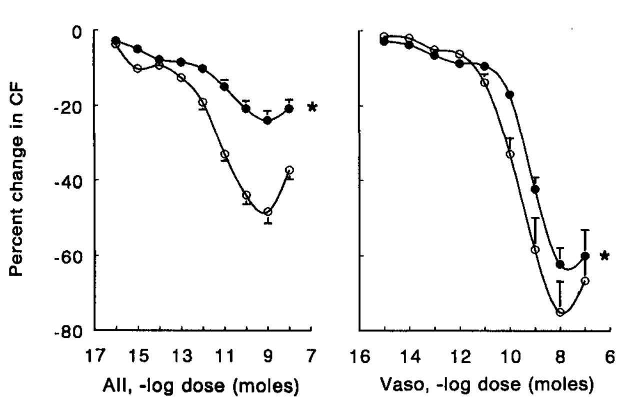

FIG. 4. Effects of bolus injections of angiotensin II (AII, left panel) and vasopressin (VASO, right panel) on coronary flow (CF) in isolated perfused rat hearts in the presence of 0.01 µM sodium nitroprusside (○) or 1 µM NDGA (●). Results are means ± S.E.M. of seven to eight experiments. Asterisks indicate significant ($p < 0.05$) difference between drug and SNP.

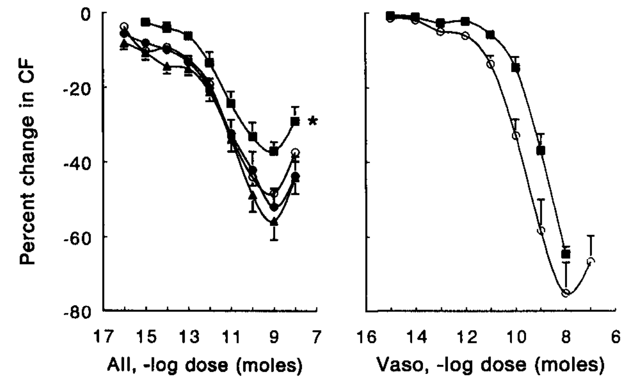

FIG. 6. Effects of bolus injections of angiotensin II (AII, left panel) and vasopressin (VASO, right panel) on coronary flow (CF) in isolated perfused rat hearts in the presence of 0.01 µM sodium nitroprusside (○), 0.1 µM FPL 55712 (●), 0.3 µM FPL 55712 (▲) or 0.3 µM MK-886 (■). Results are means ± S.E.M. of five to eight experiments. Asterisk indicates significant ($p < 0.05$) difference between drug and SNP.

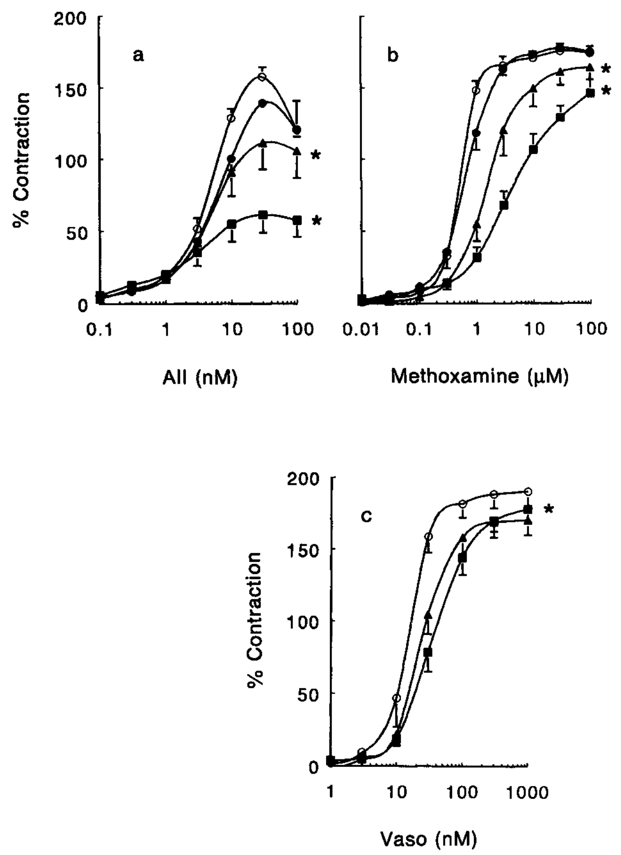

FIG. 7. Concentration-response curves of angiotensin II (AII) (a), methoxamine (b) and vasopressin (VASO) (c) in the isolated rat aorta in the absence of endothelium after incubation for 30 min with ethanol 0.1% (○), 1 μM NDGA (●), 3 μM NDGA (▲) or 10 μM NDGA (■). Results are means ± S.E.M. of six to seven experiments. The asterisks indicate significant ($p < 0.05$) difference between NDGA and ethanol pretreated aortic rings.

there was no change in maximal effect (Fig. 7c). The lipoxygenase inhibitor had no effect on the baseline tension of these rings (data not shown).

*Effects of indomethacin and AA 861 on angiotensin II-, and methoxamine-induced contractions in aortic rings without endothelium.* AA 861 (10 μM) and indomethacin (10 μM) had no effect on the contractile responses to both angiotensin II and methoxamine (Table 2).

*Effects of NDGA on angiotensin II-, and methoxamine-induced contractions in aortic rings with endothelium in the presence of L-NAME.* In this set of experiments, angiotensin II and methoxamine induced concentration dependent contractions in aortic rings with endothelium with a maximum of 78.0 ± 9.9% (eleven rings, six rats) and 199.0 ± 10.4% (eleven rings, six rats) respectively (Fig. 8, left panels).

L-NAME 100 μM significantly increased the contractions to angiotensin II (maximum, 187.8 ± 12.2%, eleven rings, six rats) and methoxamine (maximum, 209.4 ± 9.5%, twelve rings, six rats) (Fig. 8, left panels).

In the presence of L-NAME a second concentration–response curve was constructed for angiotensin II and methoxamine after incubation with either 0.1% ethanol (control) or 10 μM NDGA. In contrast to methoxamine, the maximal effect to angiotensin II of the second concentration–response curve was significantly lower compared to the first concentration–response curve. The lipoxygenase inhibitor significantly attenuated the maximal contraction to angiotensin II and shifted the concentration–response curve of methoxamine to the right (Fig. 8, right panels).

*Effects of losartan on angiotensin II-induced contractions in aortic rings without endothelium and with endothelium in the presence of L-NAME.* Incubation of denuded aortic rings with losartan (10 nM, 100 nM and 1 μM) shifted the concentration–response curve to angiotensin II to the right in a concentration dependent manner. With the highest concentration of losartan (1 μM) the angiotensin II-induced contractions (up to 300 nM angiotensin II) were virtually abolished. Similar effects were seen in intact aortic rings in the presence of 100 μM L-NAME (data not shown). The $pA_2$-value calculated for losartan in rat aorta (with and without endothelium) was 8.2.

### Discussion

The objective of this study was to determine whether arachidonate metabolites play a role in the vascular effects of angiotensin II in rats.

Table 2. Effect of 10 μM AA 861 and 10 μM indomethacin (INDO) on angiotensin II and methoxamine induced contractions in aortic rings without endothelium

| | Angiotensin II | | Methoxamine | |
|---|---|---|---|---|
| | $pD_2$ | $E_{max}$ (%) | $pD_2$ | $E_{max}$ (%) |
| Saline | 8.76 ± 0.03 (5) | 192.2 ± 10.5 (5) | 6.25 ± 0.04 (5) | 215.6 ± 16.9 (5) |
| AA 861 | 8.84 ± 0.19 (5) | 175.4 ± 11.1 (5) | 6.35 ± 0.07 (6) | 201.7 ± 9.0 (6) |
| INDO | 8.50 ± 0.12 (5) | 153.2 ± 22.3 (5) | 6.23 ± 0.08 (6) | 189.8 ± 5.3 (6) |

Between brackets, number of experiments.

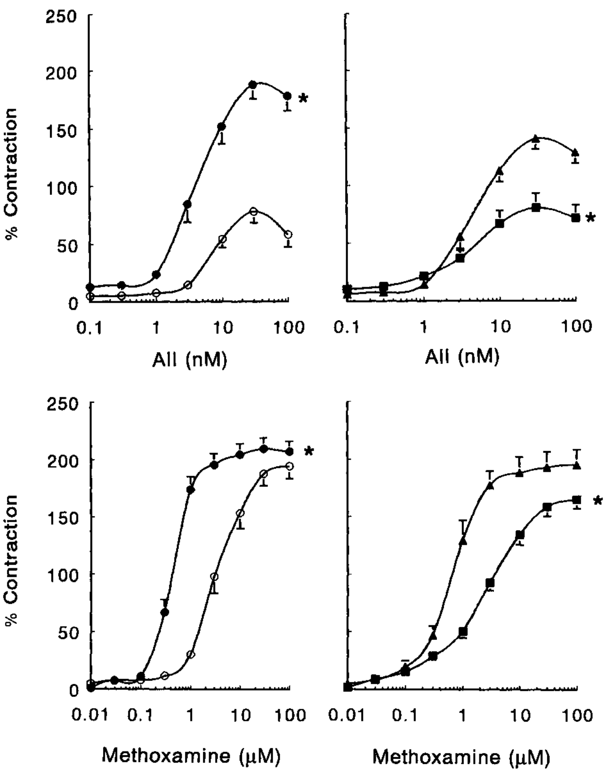

FIG. 8. Concentration–response curves of angiotensin II (AII) (upper panels) and methoxamine (lower panels) in the isolated rat aorta in the presence of endothelium. Left panels: first concentration–response curves after incubation for 30 min with saline (○) or 100 μM L-NAME (●). Right panels: second concentration–response curves after incubation for 30 min with 100 μM L-NAME and 0.1% ethanol (▲) or 100 μM L-NAME and 10 μM NDGA (■). Results are means ± S.E.M. of ten to twelve rings of six rats (experiments). The asterisks indicate significant ($p < 0.05$) difference between curves.

From our results we can conclude that: (1) in the coronary system of the rat, part of the angiotensin II-induced decreases in coronary flow are mediated by lipoxygenase products and not by cyclo-oxygenase products; (2) these lipoxygenase products might be derived from the 5-lipoxygenase pathway of arachidonate metabolism but are probably not leukotrienes; and (3) endothelium independent lipoxygenase products (not cyclo-oxygenase products) mediate part of the contractile responses to angiotensin II and to a lesser extent to methoxamine and vasopressin in the isolated rat aorta.

In the isolated perfused heart and aorta, angiotensin II acted via the $AT_1$ receptor as was shown by the inhibition by losartan.[18] We found that losartan reduced the angiotensin II-induced decreases in coronary flow and angiotensin II-induced contractions in the aorta, in a concentration dependent manner. In the presence of losartan $10^{-5}$ M, the angiotensin II effect is almost abolished in the heart. However, we did not perform experiments with a specific $AT_2$-receptor antagonist and therefore cannot exclude a (minimal) contribution of the $AT_2$-receptor to the effects of angiotensin II. The $pA_2$ value of losartan in the heart and aorta was 8.5 and 8.2 respectively. These values are similar to $pA_2$ values reported for losartan in various tissues of a number of species (e.g. rabbit aorta, rat portal vein)[18] indicating that the receptor for angiotensin II in these tissues is the same.

Several studies have demonstrated that arachidonate metabolites of the lipoxygenase pathway participate in angiotensin II dependent mechanisms, e.g. angiotensin II-induced release of neutrophil chemoattractant substance,[10] angiotensin II-induced stimulation of aldosterone secretion,[11,12] angiotensin II-induced inhibition of renin release,[13] angiotensin II-induced vasoconstriction[15,16,19] and angiotensin II-induced stimulation of cell growth.[9,14,20]

In this study, pretreatment of rats with 4 mg/kg dexamethasone 5.5 h prior to testing, significantly suppressed the maximal decreases in coronary flow to angiotensin II and vasopressin. Glucocorticoids like dexamethasone stimulate the synthesis of proteins that inhibit phospholipase $A_2$, and thus block one of the major enzymes that release arachidonic acid from the phospholipid pools.[21] The effect of dexamethasone on angiotensin II and vasopressin induced decreases in coronary flow suggests that arachidonate metabolites are involved in both angiotensin II and vasopressin-induced constrictions.

NDGA had a marked inhibitory effect on the angiotensin II-induced decreases in coronary flow and inhibited the angiotensin II-induced contractions in the aorta in a concentration dependent manner, while the cyclo-oxygenase inhibitor indomethacin had no effect in both tissues. NDGA, which is known to be a dual cyclo-oxygenase and lipoxygenase inhibitor[22] as well as an anti-oxidant, has been reported to have various nonspecific effects. Inhibition of endothelium-dependent relaxations[23] and of kinin and noradrenaline-induced contractions,[24] possibly related to the inhibition of the transmembrane calcium influx,[25] have been described. However, based on our results we have reason to believe that lipoxygenase products (not cyclo-oxygenase products) mediate at least part of the angiotensin II-induced constrictions, since in the heart several inhibitors acting at different levels of the arachidonate metabolism (NDGA, dexamethasone, MK-886) significantly reduced angiotensin II-induced decreases in coronary flow. Furthermore, we have shown that NDGA inhibited the contractions to angiotensin II in a concentration dependent manner in the aorta.

The exact lipoxygenase pathway (5-, 12-, or 15-lipoxygenase) cannot be determined from our results. The leukotriene synthesis inhibitor MK-886 significantly reduced the maximal decrease in coronary flow induced by angiotensin II in the heart. In contrast, in both aorta and heart, the 5-lipoxygenase inhibitor AA 861 had no effect on the responses to angiotensin II. MK-886 inhibits the membrane translocation of 5-lipoxygenase by an interaction with the 5-lipoxygenase activating protein and subsequently prevents the activation of 5-lipoxygenase.[26–28] MK-886 is selective for the 5-lipoxygenase pathway of arachidonate acid metabolism since it has no effect on 12-lipoxygenase or cyclo-oxygenase,[26] whereas AA 861 can also inhibit 12-hydroxyeicosatetraenoic acid (12-HETE) formation.[29] On the basis of the selectivity of MK-886, we suggest that the 5-lipoxygenase pathway is the candidate that most likely mediates decreases in coronary flow induced by angiotensin II.

The leukotriene antagonist FPL 55712 did not change the angiotensin II-induced decreases in coronary flow, indicating that the end products from the 5-lipoxygenase pathway, the leukotrienes, are not involved in angiotensin II-induced decreases in coronary flow, leaving 5-hydroperoxyeicosatetraenoic acid (5-HPETE) and 5-hydroxyeicosatetraenoic acid (5-HETE) as potential mediators of the angiotensin II-induced effects in the isolated perfused rat heart.

Saito *et al.*[30] also suggested that in cultured rat vascular smooth muscle cells angiotensin II induced constrictions are mediated by H(P)ETEs. However, in contrast with our results and in accordance with those of other investigators with respect to smooth muscle cell growth[9] and steroidogenesis,[11,14] Saito *et al.*[30] emphasized the role of 12H(P)ETE. Further studies are necessary to determine which of these two pathways, the 5-, or the 12-lipoxygenase pathway is predominant under specified conditions.

Indomethacin alone had no effect on angiotensin II-induced constrictions in both aorta and heart. The lack of effect for indomethacin on angiotensin II-induced contractions in rat aorta was also shown in other studies.[31] However, in the presence of AA 861 we observed a marked inhibitory effect of indomethacin on angiotensin II-induced effects in the isolated perfused heart. In guinea-pig hearts indomethacin induced shunting of the arachidonate metabolism through the lipoxygenase pathway.[32] Such a mechanism might enhance the synthesis of lipoxygenase products leading to e.g. 5-HPETEs or 5-HETEs when indomethacin is present.

The inhibitory effect of NDGA was not selective for angiotensin II. Vasopressin-induced decreases in coronary flow and methoxamine- and vasopressin-induced contractions in the aorta were also attenuated by NDGA; however, to a much lesser extent compared to angiotensin II. Therefore, for vasopressin and methoxamine, lipoxygenase products may also be involved in (coronary) constrictions. The effects of angiotensin II in aortic rings (denuded) were characterized by a marked reduction of the maximal effect, while for inhibition of the methoxamine and vasopressin contractions a rightward shift of the concentration–response curve was observed and no effect or a minor effect on the maximal contractions was seen. In accordance with our results are observations in aorta strips of the rabbit where 10 μM NDGA caused a 10% depression of the noradrenaline-induced tension and AA 861 (up to 30 μM) had no effect.[24] Others have demonstrated that the constrictor response to a maximal concentration of norepinephrine was not affected by the lipoxygenase inhibitor baicaleine,[16] and this is compatible with our results. Additional studies are required to determine whether the different profiles of NDGA inhibition for angiotensin II and methoxamine/vasopressin are related to differences in mechanism of action of the agonists. It has been shown that 10 μM NDGA inhibits the stimulated influx of extracellular calcium in rabbit neutrophils, without affecting cellular calcium redistribution.[25] This may contribute to the inhibitory action by NDGA at 10 μM on agonist-induced arterial contraction. In rat aortic rings, however, we already observed an inhibitory effect of NDGA at 3 μM.

NDGA (10 μM) inhibited both angiotensin II- and methoxamine-induced contractions in aortic rings with (in the presence of L-NAME) and without endothelium, indicating that lipoxygenase products that mediate angiotensin II- and methoxamine-induced contractions do not originate from the endothelium.

NDGA, AA 861, MK-886 and FPL 55712 induced a significant increase in baseline coronary flow suggesting that rat coronary flow is controlled in part by endogenously produced leukotrienes and perhaps other lipoxygenase products. In accordance, several investigators have found that AA 861, FPL 55712 and NDGA increased baseline coronary flow in perfused rat and guinea pig hearts.[33,34] A role for cyclo-oxygenase products in the regulation of rat coronary flow is not likely, since indomethacin did not affect baseline coronary flow in rats. We used control groups with SNP in sufficient concentration to reach a baseline coronary flow that was equal to the baseline coronary flow in the drug groups. To our knowledge none of the reported arachidonic acid metabolites operate through the same mechanism as SNP, i.e. elevation of cGMP production. In addition, we performed some pilot experiments with NDGA in the presence of 10 nM SNP. In those experiments the angiotensin II-induced decreases in CF were not significantly different from the decreases in CF in the presence of NDGA alone, indicating that SNP did not interfere with the effect of NDGA.

In summary, lipoxygenase products and not cyclo-oxygenase products mediate at least part of the angiotensin II-induced decreases in coronary flow in the isolated perfused heart of the rat. These products also play a small role in the constrictions to vasopressin in the isolated rat heart. The lipoxygenase products involved in the effects of angiotensin II might be derived from the 5-lipoxygenase pathway of arachidonate metabolism but are probably not leukotrienes. Furthermore, endothelium independent lipoxygenase products (not cyclo-oxygenase products) mediate part of the contractile responses to angiotensin II and to a lesser extent to methoxamine and vasopressin in the isolated rat aorta. Therefore, we can conclude that intermediary (endothelium-independent) lipoxygenase products other than leukotrienes play a role in the vascular effects of angiotensin II in the rat. Experiments with 5-HPETE and/or 5-HETE to study whether the effects of these mediators are comparable with those of angiotensin II are an interesting subject for further investigation.

### References

1. Dzau J. Vascular renin–angiotensin system and vascular protection. *J Cardiovasc Pharmacol* 1993; **22(Suppl 5):** S1–S9.
2. Lindpaintner K, Ganten D. The cardiac renin–angiotensin system. An appraisal of present experimental and clinical evidence. *Circ Res* 1991; **68:** 905–921.
3. Lindpaintner K, Jin M, Wilhelm MJ *et al.* Intracardiac generation of angiotensin and its physiologic role. *Circulation* 1988; **77(Suppl I):** I18–I23.
4. Mebazaa A, Chevalier B, Mercadier JJ, Echter E, Rappaport L, Swynghedauw B. A review of the renin–angiotensin system in the normal heart. *J Cardiovasc Pharmacol* 1989; **14(Suppl 4):** S16–S20.
5. Becker RH, Linz W, Scholkens BA. Pharmacological interference with the cardiac renin–angiotensin system. *J Cardiovasc Pharmacol* 1989; **14(Suppl 4):** S10–S15.
6. Kaski JC, Rosano G, Gravielides S, Chen L. Effects of angiotensin-converting enzyme inhibition on exercise-induced angina and ST segment depression in patients with microvascular angina. *J Am Coll Cardiol* 1994; **23(3):** 652–657.
7. Baker KM, Aceto JF. Angiotensin II stimulation of protein synthesis and cell growth in chick heart cells. *Am J Physiol* 1990; **259:** H610–H618.
8. Griendling KK, Tsuda T, Berk BC, Alexander RW. Angiotensin II stimulation of vascular smooth muscle. *J Cardiovasc Pharmacol* 1989; **14(Suppl 6):** S27–S33.
9. Natarajan R, Gonzales N, Lanting L, Nadler J. Role of the lipoxygenase pathway in angiotensin II-induced vascular smooth muscle cell hypertrophy. *Hypertension* 1994; **23(Suppl I):** I142–I147.
10. Farber HW, Center DM, Rounds S. Bovine and human endothelial cell production of neutrophil chemoattractant activity in response to components of the angiotensin system. *Circ Res* 1985; **57:** 898–902.
11. Nadler JL, Natarajan R, Stern N. Specific action of the lipoxygenase pathway in mediating angiotensin II-induced aldosterone synthesis in isolated adrenal glomerulosa cells. *J Clin Invest* 1987; **80:** 1763–1769.
12. Natarajan R, Stern N, Hsueh W, Do Y, Nadler J. Role of the lipoxygenase pathway in angiotensin II-mediated aldosterone biosynthesis in human adrenal glomerulosa cells. *J Clin Endocrinol Metab* 1988; **67:** 584–591.
13. Antonipillai L, Nadler J, Horton R. Angiotensin feedback inhibition on renin is expressed via the lipoxygenase pathway. *Endocrinology* 1988; **122:** 1277–1281.
14. Natarajan R, Gonzales N, Hornsby PI, Nadler J. Mechanism of angiotensin II-induced proliferation in bovine adrenocortical cells. *Endocrinology* 1992; **131:** 1174–1180.
15. Bell-Quilley CP, Lin YR, Hilchey SD, Drugge ED, McGiff JC. Renovascular actions of angiotensin II in the isolated kidney of the rat: Relationship to lipoxygenases. *J Pharmacol Exp Ther* 1993; **267(2):** 676–682.
16. Stern N, Golub M, Nozawa K, *et al.* Selective inhibition of angiotensin II-mediated vasoconstriction by lipoxygenase blockade. *Am J Physiol* 1989; **257:** H434–H443.
17. Vleeming W, van der Wouw PA, van Rooij HH, Wemer J, Porsius AJ. *In vitro* method for measurement of cardiac performance and responses to inotropic drugs after experimentally induced myocardial infarction in the rat. *J Pharmacol Methods* 1989; **21:** 95–102.
18. Rhaleb N, Noureddine R, Nantel F, D'Orléans-Juste P, Regoli D. DuP 753 is a specific antagonist for the angiotensin receptor. *Hypertension* 1991; **17:** 480–484.
19. Müller-Schweinitzer E, Olea Baza I. Pharmacological evidence for the existence of a local renin–angiotensin system in porcine interlobar renal arteries. *Br J Pharmacol* 1990; **101:** 89–92.
20. Natarajan R, Gu J, Rossi J, *et al.* Elevated glucose and angiotensin II increase 12-lipoxygenase activity and expression in porcine aortic smooth muscle cells. *Proc Natl Acad Sci USA* 1993; **90:** 4947–4951.
21. Blackwell GJ, Carnuccio R, Di Rossa M, Flower RJ, Parente L, Persico P. Macrocortin: a polypeptide causing the anti-phospholipase effect of glucocorticoids. *Nature* 1980; **287:** 147–149.
22. Piomelli D, Greengard P. Lipoxygenase metabolites of arachidonic acid in neuronal transmembrane signalling. *Trends in Pharmacol Science* 1990; **11:** 367–373.
23. Furchgott RF. The role of endothelium in the responses of vascular smooth muscle to drugs. *Ann Rev Pharmacol Toxicol* 1984; **24:** 175–197.
24. Deblois D, Bouthillier J, Marceau F. Effect of glucocorticoids, monokines and growth factors on the spontaneously developing responses of the rabbit isolated aorta to des-Arg$^9$-bradykinin. *Br J Pharmacol* 1988; **93:** 969–977.
25. Naccache PH, Showell HJ, Becker EL, Sha'afi RI. Pharmacological differentiation between the chemotactic factor induced cellular calcium redistribution and transmembrane calcium influx in rabbit neutrophils. *Biochem Biophys Research Commun* 1979; **89(4):** 1224–1230.
26. Gillard J, Ford Hutchinson AW, Chan C, *et al.* L-663,536 (MK-886) (3-[1-(4-chlorobenzyl)-3-*t*-butyl-thio-5-isopropylindol-2-yl]-2,2-dimethylpropanoic acid), a novel, orally active leukotriene biosynthesis inhibitor. *Can J Physiol Pharmacol* 1989; **67:** 456–464.
27. Miller DK, Gillard JW, Vickers PJ, *et al.* Identification and isolation of a membrane protein necessary for leukotriene production. *Nature* 1990; **343:** 278–281.
28. Rouzer CA, Ford Hutchinson AW, Morton HE, Gillard JW. MK886, a potent and specific leukotriene biosynthesis inhibitor blocks and reverses the membrane association of 5-lipoxygenase in ionophore-challenged leukocytes. *J Biol Chem* 1990; **265:** 1436–1442.
29. Ashida Y, Saijo T, Kuriki H, Makino H, Terao S, Maki Y. Pharmacological profile of AA-861, a 5-lipoxygenase inhibitor. *Prostaglandins* 1983; **26(6):** 955–972.
30. Saito F, Hori MT, Ideguchi Y, *et al.* 12-Lipoxygenase products modulate calcium signals in vascular smooth muscle cells. *Hypertension* 1992; **20:** 138–143.
31. Gruetter CA, Ryan ET, Lemke SM, Bailly DA, Fox MK, Schoepp DD. Endothelium-dependent modulation of angiotensin II-induced contraction in blood vessels. *Eur J Pharmacol* 1988; **146:** 85–95.
32. Aehringhaus U, Dembinska-Kiec A, Peskar BA. Effects of exogenous prostaglandins on the release of leukotriene C4-like immunoreactivity and on coronary flow in indomethacin-treated anaphylactic guinea-pig hearts. *Naunyn Schmiedebergs Arch Pharmacol* 1984; **326:** 368–374.
33. Vleeming W, van Rooij HH, Wemer J, Porsius AJ. Characterization and modulation of antigen-induced effects in the isolated heart of the rat. *J Cardiovasc Pharmacol* 1991; **18:** 556–565.
34. Aehringhaus U, Peskar BA, Wittenberg HR, Wolbling RH. Effect of inhibition of synthesis and receptor antagonism of SRS-A in cardiac anaphylaxis. *Br J Pharmacol* 1983; **80:** 73–80.

ACKNOWLEDGEMENTS. We thank Harry van Lierop and Harriët Tomassen for excellent technical assistance.

**Received 2 August 1995; accepted in revised form 5 September 1995**
